# Data-driven prediction in dynamical systems: recent developments

**DOI:** 10.1098/rsta.2021.0213

**Published:** 2022-08-08

**Authors:** Amin Ghadami, Bogdan I. Epureanu

**Affiliations:** Department of Mechanical Engineering, University of Michigan, Ann Arbor, MI, USA

**Keywords:** data-driven prediction, model discovery, dynamical systems

## Abstract

In recent years, we have witnessed a significant shift toward ever-more complex and ever-larger-scale systems in the majority of the grand societal challenges tackled in applied sciences. The need to comprehend and predict the dynamics of complex systems have spurred developments in large-scale simulations and a multitude of methods across several disciplines. The goals of understanding and prediction in complex dynamical systems, however, have been hindered by high dimensionality, complexity and chaotic behaviours. Recent advances in data-driven techniques and machine-learning approaches have revolutionized how we model and analyse complex systems. The integration of these techniques with dynamical systems theory opens up opportunities to tackle previously unattainable challenges in modelling and prediction of dynamical systems. While data-driven prediction methods have made great strides in recent years, it is still necessary to develop new techniques to improve their applicability to a wider range of complex systems in science and engineering. This focus issue shares recent developments in the field of complex dynamical systems with emphasis on data-driven, data-assisted and artificial intelligence-based discovery of dynamical systems.

This article is part of the theme issue 'Data-driven prediction in dynamical systems'.

## 1. Introduction

Dynamical systems play a key role in deepening our understanding of the physical world. In dynamical system analysis, the need for forecasting the future state of a dynamical system is a critical need that spans across many disciplines ranging from climate, ecology and biology to traffic and finance [1–5]. Predicting complex dynamics is the foundation of the subsequent design, control and improvement of physical systems.

Conventionally, prediction in dynamical systems is done by first creating a model of the system, derived from first principles or experimental data. In this approach, the mathematical structure of a model is established, and observational data are used to inform it [6–8]. However, the increased complexity of systems poses challenges to formalizing models, even roughly accurate, for modern dynamical systems. Physical phenomena are very complex in many real-world systems, and they are not amenable to classical model-based approaches due to the computational size of these problems and lack of accurate models. In those cases, it is tempting to resort to data-driven approaches to model, analyse, and predict dynamical system entirely from data.

The problem of characterizing and forecasting in dynamical systems dates back to the 1980 s and 1990 s when dynamicists developed methods to analyse time series recorded from nonlinear and chaotic systems [9–12]. These frameworks rely on the pioneering work of Takens [13], introducing the concept of reconstruction of the state space of the system from which the data are sampled, and then study the system in this new representation. This development was the basis for the subsequent development of non-parametric methods for time series forecasting [14–21].

Thanks to recent improvements in storage and computing power, more experimental, observational and simulated data are now available. Consequently, data-driven approaches to study dynamical systems have attracted increasing attention in recent years and now can tackle previously unattainable challenges in modelling and prediction of complex systems in a variety of fields [22–24]. Extracting dynamical behaviours from data have been one of the main research goals in recent years [25–30]. This line of research has focused on analysis of data recorded from system dynamics to reveal much of the desired properties of the system at hand. Examples include data-driven identification of transfer operators and their eigenvalues, eigenfunctions and eigenmodes [31,32], data-driven identification of coherent structures in dynamical systems [33–38], and bifurcation forecasting methods for data-driven nonlinear stability analysis of dynamical systems [39–44].

The generation of physical and mathematical models from experimental data is another field of study that has risen to prominence as computers have become faster, more powerful and more affordable [45]. Today's systems are complex and large, often with a massive number of unknown parameters that makes prediction and design of control policies to achieve a desired behaviour a challenge. As a result, developing simple and tractable data-driven system identification methods via a limited number of recorded observations has been the motivation of recent research. These methods focus on the discovery of dynamical systems from high-dimensional data [46–55] and making predictions of system dynamics based on the identified model. Examples include data-driven identification methods based on nonlinear regression [56], empirical dynamic modelling [57], normal form methods [58], nonlinear Laplacian spectral analysis [59], eigensystem realization algorithms [60], dynamic mode decomposition (DMD) [52,54] and artificial neural networks [61,62]. For cases where a latent differential equation is believed to exist, data-driven discovery techniques have been developed to identify the best fit to data based on pre-defined equations [63–65]. More recent progress in compressed sensing and sparse regression in a library of candidate models has also been proposed to identify dynamical systems [66–70] and partial differential equations [55] to provide the best trade-off between model complexity and predictive accuracy.

Despite the widespread availability of measurement and simulation data, the sheer size, complexity and dimensionality of modern datasets pose their own challenges. Accurate and computationally tractable prediction in dynamical systems requires development of low-dimensional models that are fast to solve but that approximate well the underlying high-resolution dynamics. This has led to a growing need for dimensionality reduction and reduced-order modelling techniques, and made these an inseparable part of data-driven prediction algorithms [71]. Based on data collected either from simulation or from experiments, reduced-order techniques identify an alternate space having comparatively a smaller number of active variables and/or coordinates where the dynamical evolution is tractable. The literature on reduced-order modelling is quite mature. The most celebrated model reduction technique is the proper orthogonal decomposition (POD) [72–76] and its various variants, such as local and global POD methods [77,78], adaptive POD methods [79,80], double POD methods [81,82] and gappy POD method [83] to name a few. In addition to POD, there are a variety of other methods developed for an improved accuracy and computational efficiency motivated by different applications. Examples include balanced truncation [84–86], balanced POD [87], Krylov subspace methods [88], missing point estimation [89] and trajectory-based optimization for oblique projections [90]. Dynamic reduced-order models are another development in this field that exploit the opportunity presented by dynamic sensor data and adaptively incorporate sensor data during the online phase [91]. One common feature among these approaches is that, using numerical simulations or experimental measurements, they identify a few coordinates with high variance or energy that significantly influence the dynamics. If governing equations are available, Galerkin or Petrov-Galerkin projection [72,74,92,93] are then be used to create a reduced model using the identified few modes that contain the majority of the system's energy. Recently, fully data-driven techniques, particularly DMD methods [52,54,94], supported by Koopman operator theory [95,96], have been introduced as both system identification and model reduction technique and received a growing attention in a variety of fields. These algorithms were proposed to extract dynamically relevant features from time-resolved experimental or numerical data without explicit knowledge of the dynamical operator. These algorithms create a best-fit linear operator to advance spatio-temporal snapshots forward in time. In addition, advances in machine learning techniques have resulted in the use of autoencoders that aim to learn the identity mapping through a dimensionality reduction procedure comprising data compression and subsequent recovery [97–100]. Such approaches have made the problem of nonlinear reduced-order modelling tractable, which was otherwise a challenging task.

With the aid of more powerful hardware, computational models and algorithms, recent research on data-driven prediction has been heavily influenced by machine learning algorithms, particularly neural networks [101,102]. These methods are used to make predictions in all kinds of time series [103–106], and achieved a significant success thanks to the abundance of data. In addition, the combination of traditional methods and deep learning techniques received significant attention and resulted in breakthroughs [107–111]. The use of neural networks for identification and prediction of dynamical systems dates back to more than three decades ago [112,113]. One of the most important early results in the field of predicting dynamics using neural network came from Herbert Jaeger & Harald Haass [108] where a particular kind of neural network, called a reservoir computer, used to forecast the Mackey–Glass dynamical system. In recent years, a variety of innovative machine learning techniques for system identification and prediction have been proposed. Among those, recurrent neural networks (RNNs) [114,115] and long short-term memory networks (LSTM) [116–118] are most commonly used for time series forecasting. Other approaches include data-driven discovery using deep neural networks (DNNs) [61], ordinary differential equation networks (ODENet) [107], deep residual learning [110], reservoir computing [119], convolutional neural networks [120], deep Koopman methods [121,122] and recently introduced deep operator networks (DeepONet) for learning continuous nonlinear operators from data [111]. These approaches provide a neural network model that mimics system dynamics when little or no physical knowledge is known but a large amount of data are available. However, it is likely that the learned models do not generalize well on the region where training data are scarce or do not exist. This challenge motivated the development of another group of methods called physics-informed data-driven approaches, which model governing equations by encoding differential equations describing physical processes into neural networks loss function to penalize the network if do not obey the physics [109,123]. By exploiting fundamental physical laws, the amount of data needed to obtain good performance is much less than for pure data-driven methods, and neural networks approximately follow physical laws after training. Subsequent development of these approaches resulted in the development of network architectures that incorporate assumptions about the nature and underlying physical laws in the neural network topology for an improved training for particular physical systems. Examples include Hamiltonian neural networks [124,125] and symplectic networks (SympNets) [126] for learning Hamiltonian systems, and deep Lagrangian networks [127] for learning Lagrangian systems while ensuring physical plausibility.

Energized by the success of these generic methods, interest has grown for incorporating data-driven methods to tackle previously unattainable challenges in modelling and prediction of dynamical systems in a variety of applications. Examples include but are not limited to health sciences [128–132], biology [133–135], epidemiology and infectious disease [136,137], ecology and climate [138–142], financial markets [143], fluid dynamics [96,144–146], aeroelasticity [147–149], solid mechanics and materials [150–153], energy [154–156], transportation [157–161] and heat transfer [162–164]. Data-driven prediction methods have also offered a solution to the formidable challenge of predicting catastrophic events in a variety of complex systems. Recent studies have shown that features extracted from data can be used to predict critical transitions [165] and extreme events [166] in the dynamics of a variety of complex systems, including aeroelastic systems [39,40,44,167,168], ecological systems [51,169–175], epidemiological systems [176–179], traffic flow systems [158,180] and fluid flows [166,181–183].

In the light of the current abundance of sensor data and advances in data-driven and machine learning techniques, it is inevitable that data-driven techniques will dominate the future of dynamical systems to tackle the modelling, prediction and control challenges facing science and engineering. While data-driven prediction methods have made great strides, it is still necessary to develop new and improved techniques to enhance their effectiveness and applicability to a wider range of complex systems in nature and engineering. This special issue provides a platform to share recent developments in the field of data-driven, data-assisted and artificial intelligence-based discovery of dynamical systems. In this focus issue, 15 papers that are addressing timely topics in the field are included. In the following, a brief overview of each paper is presented.

## 2. The general content of the issue

Data-driven dimensionality reduction techniques are a crucial part of analysis in large dimensional complex dynamical systems. Traditional linear dimensionality reduction techniques are not suitable for problems whose underlying governing equations are characterized by local solution features that evolve with time. To address this challenge, localization-based reduced-order modelling techniques have been developed in which multiple local approximation spaces are tailored to different regions of the state-space. Existing approaches, however, require access to high-fidelity models or codes to compute the projected reduced-order operators. That limits the applicability of these approaches. Geelen & Willcox [184] present data-driven learning of localized reduced models combining the state-of-the-art localization techniques with a non-intrusive model reduction formulation, leading to a flexible framework for reduction of large-scale nonlinear problems. The proposed approach is particularly important for reduced models of nonlinear systems of partial differential equations, where the solution may be characterized by different physical regimes or exhibit high sensitivity to parameter variations.

POD-based reduced-order modelling techniques are effective for systems in which the most reachable and the most observable directions are aligned, which is not the case in many systems including in most transport problems. Balance truncation methods can offer a solution to this challenge in standard projection techniques. Application of balance truncation in stiff systems with lightly damped (slowly decaying) impulse response is challenging due to triggering instabilities and intensifying sensitivity to sampling properties in the method. To address the realizability and scalability of balance truncation applied to highly stiff and lightly damped systems, Rezaian *et al*. [185] introduce a non-intrusive data-driven method for balancing discrete-time systems via the eigensystem realization algorithm (ERA). The advantage of ERA for balancing transformation makes full-state outputs tractable and enables balancing despite stiffness, by eliminating computation of balancing modes and adjoint simulations.

Developing reduced-order models for mechanical systems is also an important topic in engineering. For instance, the need to capture amplitude-dependent properties and competing steady-state solutions are increasingly important to identify as highlighted in experiments of nonlinear mechanical vibrations. While data-driven model reduction techniques are well established for linearizable mechanical systems, general approaches to reducing nonlinearizable systems with multiple coexisting steady states have been unavailable. Cenedese *et al*. [186] discuss a new data-driven reduced-order modelling approach in the context of mechanical vibrations, which is dynamics-based rather than physics-informed. Built on the recent theory of spectral submanifolds (SSMs), this approach identifies very low-dimensional, sparse models over different time scales by restricting the full system dynamics to a nested family of attractors. This approach constructs normal forms on attracting SSMs, which are the smoothest nonlinear continuation of spectral subspaces of the linearized dynamics.

DMD techniques are data-driven reduced-order modelling and system identification methods that have been broadly used in the scientific community due to their ease of use, interpretability and adaptability. These methods provide a data-driven regression architecture for adaptively learning linear dynamics models over snapshots of temporal data. The majority of classical DMD algorithms, however, are prone to bias errors from noisy measurements of the dynamics, leading to poor model fits and unstable forecasting capabilities. Sashidhar & Kutz [187] introduce an optimized DMD method, called bagging optimized dynamic mode decomposition (BOP-DMD), by using statistical bagging methods that improves the performance of DMD methods. Unlike currently available DMD algorithms, BOP-DMD provides a stable and robust model for probabilistic or Bayesian forecasting with comprehensive uncertainty quantification metrics.

The effectiveness of reduced-order models depends on their design so they can capture the complexity of the underlying dynamics. However, identifying effective reduced-order models also depends on the information they rely on. This fundamental information in complex systems is provided by data, which is often expensive. Sapsis & Blanchard [188] introduce a criterion based on a Gaussian process regression (GPR) for the most effective selection of data or the associated experiments to generate this data to perform data-driven reduced-order modelling. In particular, an optimality condition for the selection of a new input is defined as the minimizer of the distance between the approximated output probability density function of the reduced-order model and the exact one, which is defined as the supremum over the unit sphere of the native Hilbert space for the GPR.

To identify and predict system dynamics, physics-informed identification of dynamical systems has received a growing attention in recent years. These methods aim to model governing equations by embedding physics into neural networks together with data, thereby mitigating the problem of the learned models not extending well to regions where there is a lack of training data. One approach to impose physics constraints on neural networks is by designing proper neural network architectures that obey the underlying principles without optimization processes, yet maintaining sufficient expressivity so that governing equations can be learned from data. Zhang *et al*. [189] provide a novel neural network surrogate model, called a GENERIC formalism informed neural network (GFINN), allowing flexible ways of leveraging available physical information into neural networks. The imposed physics is based on the GENERIC (General Equation for Non-Equilibrium Reversible-Irreversible Coupling) formalism, providing conditions interpreted as the first and second principles of thermodynamics. This approach can handle inference and prediction of deterministic and stochastic irreversible thermodynamic processes using structured neural networks, while strictly preserving the thermodynamics constraints described by the GENERIC formalism at the same time.

When no knowledge about a physical system is available, deep learning methods require large amounts of data for generalized solutions, which may pose a challenge for identification and prediction of many real-world dynamical systems. Learning spatio-temporal processes purely using data is an example of such challenges. Saha & Mukhopadhyay [190] address the problem of predicting complex, nonlinear spatio-temporal dynamics when data are recorded at irregularly spaced sparse spatial locations. The proposed method does not assume any specific physical representation of the underlying dynamical system, and is applicable to spatio-temporal dynamical systems involving continuous state variables. The proposed approach is based on radial basis function (RBF) collocation method which is often used for meshfree solution of partial differential equations. This framework enables unravelling the observed spatio-temporal function and learning the spatial interactions among data sites on the RBF-space. The learned spatial features are then used to perform predictions in future time steps.

Most existing data-driven systems identification techniques heavily depend on the quality of the available observations and learning dynamics from noisy and irregular observations is still a challenge. Bhouri & Perdikaris [191] present a machine learning framework (GPNODE) for Bayesian model discovery from partial, noisy and irregular observations of nonlinear dynamical systems. The proposed method takes advantage of differentiable programming to propagate gradient information through ordinary differential equation solvers and perform Bayesian inference with respect to unknown model parameters using Hamiltonian Monte Carlo sampling and Gaussian Process priors over the observed system states.

Though there is an increasing amount of data for complex systems and methods for discovering the laws governing dynamical systems, existing techniques are focused mostly on deterministic or stochastic systems with Gaussian noise. Lu *et al*. [192] present a new data-driven approach to extract stochastic governing laws with both (Gaussian) Brownian motion and (non-Gaussian) Lévy motion from short bursts of simulation data. The normalizing flow technique is used to estimate the transition probability density function from data and approximate Lévy jump measure, drift coefficient and diffusion coefficient of a stochastic differential equation using non-local Kramers–Moyal formulae.

The closure problem in nonlinear dynamical systems is another key area in computational statistics. One of the difficulties in this statistical closure problem is the lack of training data. Qi & Harlim [193] propose a machine learning non-Markovian closure modelling framework for accurate predictions of statistical responses of turbulent dynamical systems subjected to external forcing. A unified model framework is proposed aiming to directly predict the leading-order statistical moments subjected to general external perturbations, with limited training data. In this work, motivated by practical issues in obtaining longer time series, short-time transient statistical sequences are considered for training.

Stability analysis of dynamical systems is a critical requirement in predicting the dynamics of complex systems. For systems exhibiting bifurcation instabilities, centre manifold theory in conjunction with the theory of normal forms offer generic and reduced forms for a variety of bifurcations. However, traditional methods to identify the dynamics on the centre manifold require an accurate model of the system as well as considerable algebra using the nonlinear system equations. Ghadami & Epureanu [194] present a deep learning structure that can uncover the system dynamics on the centre manifold for a class of systems prone to co-dimension one instabilities. Using random snapshots recorded from system dynamics before the onset of an instability, the method identifies whether the system is at risk of instabilities in its dynamics, and returns a closed-form model of the system dynamics on the centre manifold facilitating stability analysis of large-dimensional dynamical systems from data.

Extending the state of the art in topology-based methods for nonlinear dynamical systems, Ghalyan *et al*. [195] focus on developing a robust machine learning method that makes use of topological invariants in data manifolds for a robust pattern recognition and anomaly detection in dynamical systems. In the proposed approach, pattern recognition and anomaly detection are viewed from topological perspectives, where changes within a phase are described by topological transformations that preserve topological invariants, while changes between different phases imply changes in these topological invariants. The proposed approach is validated on models of selected chaotic dynamical systems for prompt detection of phase transitions.

Data-driven and data-assisted algorithms reduce the computation costs associated with traditional approaches in a variety of applications. The dynamics of dispersions of small particles in a fluid are an important example in many engineering and medical applications. While the dynamics of these particles can be simulated directly for a specific (sampled) dispersion by tracking each particle, distribution statistics are typically sought in applications demanding expensive simulations. Quadrature-based moment methods (QBMMs) offer a low-cost approach to address this issue. However, these methods can exhibit numerical instabilities when high-order moments are evolved. Charalampopoulos *et al*. [196] propose a data-informed conditional hyperbolic quadrature method for statistical moments. This approach addresses previous challenges by training a RNN that adjusts the QBMM quadrature to evaluate unclosed moments with higher accuracy. The method is applied to the statistics of a population of spherical bubbles oscillating in response to time-varying randomized forcing, and may also be effectively applied to dynamical systems with non-Gaussian statistics where high-order moments are of interest.

Data-driven techniques offer a solution to many challenging engineering problems. McClellan *et al*. [197] propose a two-level, data-driven, digital twin concept for the autonomous landing of aircraft. The main purpose of the proposed approach is to predict the state of an aircraft and the aerodynamic forces and moments acting on it in real-time. Unlike static lookup tables or regression-based surrogate models based on steady-state wind tunnel data, this real-time digital twin prototype allows the digital twin instance for model predictive control to be informed by a truly dynamic flight model, rather than a less accurate set of steady-state aerodynamic force and moment data points.

Numerical time-stepping algorithms to approximate solutions of nonlinear differential equations are a key in analysing dynamical systems. Time-stepping schemes are typically based on Taylor series expansions that are local in time and have a numerical accuracy determined by the step size. Many systems characterized by multiscale physics exhibit dynamics over a vast range of timescales, making numerical integration expensive. Liu *et al*. [198] introduce a data-driven time-stepper framework based on synthesizing multiple DNNs for hierarchical time-steppers (HiTSs) trained at multiple temporal scales. The proposed method explicitly takes advantage of dynamics on different scales by learning flow-maps for those different scales. The proposed approach is shown to outperform neural networks trained at a single scale, providing an accurate and flexible approach for integrating nonlinear dynamical systems.

## Data Availability

This article has no additional data.
